# Association of the MAOA promoter uVNTR polymorphism with suicide attempts in patients with major depressive disorder

**DOI:** 10.1186/1471-2350-12-74

**Published:** 2011-05-24

**Authors:** For-Wey Lung, Dong-Sheng Tzeng, Mei-Feng Huang, Ming-Been Lee

**Affiliations:** 1 Department of Psychiatry, Kaohsiung Armed Forces General Hospital, Kaohsiung, Taiwan; 2 Department of Neurology, Kaohsiung Medical University, Kaohsiung, Taiwan; 3 Department of Psychiatry, National Defense Medical Center, Taipei, Taiwan; 4 Graduate Institute of Undersea Medicine, National Defense Medical Center, Taipei, Taiwan; 5 Department of Psychiatry, Kai-Suan Psychiatric Hospital, Kaohsiung, Taiwan; 6 Department of Psychiatry, National Taiwan University College of Medicine, Taipei, Taiwan; 7 Department of Social Medicine, National Taiwan University College of Medicine, Taipei, Taiwan; 8 Director of Taiwan Suicide Prevention Center, Taipei, Taiwan

**Keywords:** MAOA u-VNTR Polymorphisms, Suicide Attempts, Major Depressive Disorder

## Abstract

**Background:**

The MAOA uVNTR polymorphism has been documented to affect the MAOA gene at the transcriptional level and is associated with aggressive impulsive behaviors, depression associated with suicide (depressed suicide), and major depressive disorder (MDD). We hypothesized that the uVNTR polymorphism confers vulnerability to MDD, suicide or both. The aim of this study was to explore the association between the MAOA uVNTR and depressed suicide, using multiple controls.

**Methods:**

Four different groups were included: 432 community controls, 385 patients with MDD who had not attempted suicide, 96 community subjects without mental disorders who had attempted suicide, and 109 patients with MDD who had attempted suicide. The MAOA uVNTR polymorphism was genotyped by a PCR technique. The symptom profiles and personal characteristics in each group were also compared.

**Results:**

The MAOA 4R allele was more frequent in males with MDD than in male community controls (χ^2 ^= 4.182, p = 0.041). Logistic regression analysis showed that, among the depressed subjects, those younger in age, more neurotic or who smoked had an increased risk of suicide (β = -0.04, p = 0.002; β = 0.15, p = 0.017; β = 0.79, p = 0.031, respectively). Moreover, among those who had attempted suicide, those younger in age, with more paternal overprotection, and more somatic symptoms were more likely to be in the MDD group than in the community group (β = -0.11, p < 0.001; β = 0.15, p = 0.026; β = 1.11, p < 0.001). Structural equation modeling (SEM) showed that nongenetic factors, such as age, paternal overprotection, and somatic symptoms, were associated with MDD, whereas depressed suicide were associated with severity of depression, personality traits, age, marital status, and inversely associated with anxiety symptoms. However, depression did not affect suicidal behavior in the community group.

**Conclusion:**

The MAOA 4R allele is associated with enhanced vulnerability to suicide in depressed males, but not in community subjects. The MAOA 4R allele affects vulnerability to suicide through the mediating factor of depressive symptoms. Further large-scale studies are needed to verify the psychopathology of the relationships among MAOA uVNTR polymorphism, symptom profiles, and suicidal behavior.

## Background

According to the results of a population-based survey, 16.3% of young adults in the general population in the USA and 25.2% of individuals with a general medical condition develop suicidal ideation [1]. In Taiwan, symptoms of depression have been reported to be one of the three major predictors related to the development of suicidal ideation in psychiatric patients [2]. It has also been reported that serious suicidal behavior might occur more readily in those patients with mental illness or psychiatric comorbidity during their lifetime. However, the relationship between psychiatric comorbidity and the risk of suicide varies with age and gender [3]. Individuals sometimes also commit suicide in the absence of psychiatric disorders [1,2]. Furthermore, Lung and Lee [2] found that different factors contribute to the risk of suicide in different groups. Depression, hostility, and feelings of inferiority are predictors of suicide in patients with psychiatric illness, whereas hostility, feelings of inferiority, and insomnia are predictors of suicide in subjects from the community [2]. This demonstrates that the factors that affect attempted suicide in psychiatric patients and in the general population might differ.

In addition to the above-mentioned proximal factors, personal characteristics, which include gender, socioeconomic status, age, education, and personality, are important factors that are associated with suicidal behavior. Some studies have found that neuroticism is associated with increased rates of suicide [4,5], whereas extraversion has a negative association [5]. Moreover, few studies of suicide have included the proximal psychological autopsy and distal predisposing factors among the factors investigated [6,7].

A family history of suicidal behavior and certain genetic factors are linked to the development of suicidal ideation [8,9]. Increasingly, studies have indicated that gene-environment interactions might play a fundamental role in the occurrence of suicide attempts [10]. Abnormalities of the serotonergic system have been found to be involved in the risk of suicide. For example, the 102C allele of the 5-HT2A serotonin receptor is associated with suicidal ideation in depressive patients, and the L/L (long/long) genotype of the 5-HTTLPR polymorphism of the serotonin transporter gene is associated significantly with completed suicide. In contrast, the S/S (short/short) genotype is found more frequently in individuals who have attempted suicide but failed, and who have further suicidal behavior, than in those who have not [11,12].

The gene for monoamine oxidase A (MAOA), which is located on chromosome Xp21-p11 [13], is involved in the serotonergic pathway, and contains a region within its promoter that is polymorphic with respect to the number of copies of a 30-base pair repeat. The alleles that have been identified for this upstream variable number of tandem repeats (uVNTR) polymorphism include alleles with 3, 3.5, 4, and 5 repeats (3R, 3.5R, 4R, and 5R) [14]. These different uVNTR variants are associated with different transcriptional activities of the MAOA promoter [15], which in turn result in different levels of expression of the MAOA gene. The allele of the MAOA uVNTR polymorphism that results in high levels of expression (high activity-related allele) has been reported to be associated in males with suicide caused by depression [16]. In addition, postmortem results have shown an increased level of MAOA activity in the hypothalamus of the brains of suicide victims with depressive disorder [17]. By contrast, in a population of healthy subjects from the community, male carriers of the low activity alleles (3R and 5R) showed lower scores on a composite measure of dispositional aggressiveness and impulsivity, and showed a greater responsiveness to serotonin in the central nervous system (CNS), than men with the high activity allele [18].

The MAOA uVNTR polymorphism has been identified as a genetic factor that can modulate the risk for depression, suicide, or both by influencing monoaminergic activity in a sexually dimorphic manner [16]. However, little is known about whether the MAOA uVNTR polymorphism confers vulnerability to major depressive disorder (MDD) or suicidal behavior for the Han Chinese population in Taiwan.

Gene-gene interactions are common in human diseases. For example, the apolipoprotein (Apo) ε2 allele is a potential confounding covariate for the MAOA uVNTR polymorphism in MDD: the high activity MAOA uVNTR alleles are associated with MDD when the Apo ε2 allele, which is a protective factor for MDD, is adjusted for [19]. Moreover, gene-environment interactions are also an important factor. For example, Lin and colleagues also identified a sex-specific interaction between the MAOA uVNTR polymorphism and MDD [19]. In addition, maltreated children who carry the high activity alleles of the MAOA uVNTR are less likely to develop antisocial problems and behaviors than maltreated children who carry the low activity alleles [20].

Therefore, in this study, we aimed to investigate the role of the different MAOA variants in relation to MDD and/or attempted suicide. We used structural equation modeling (SEM) to investigate the relationships among personality traits, and other demographic factors, in participants with MDD and those from the community who had or had not attempted suicide.

## Methods

### Sample Collection

We recruited four groups of participants: 1) controls from the community, 2) patients with MDD, 3) subjects without mental disorders who had attempted suicide recruited from the emergency room, and 4) patients with MDD who had attempted suicide. A total of 1022 participants were recruited: the community control group included 432 participants; the MDD group, 385 participants; community subjects who had attempted suicide, 96 participants; and the group of patients with MDD who had attempted suicide, 109 participants. The patients with MDD were recruited from a teaching hospital in southern Taiwan from April 2001 to March 2006. All the participants were interviewed by two senior psychiatrists and research assistants to ensure that they did or did not meet the psychiatric diagnosis of MDD, according to the *Diagnostic and Statistical Manual of Mental Disorders *[21] using the Mini-international Neuropsychiatric Interview (MINI) [22].

### Materials

Personality and symptom profiles were collected. Personality was assessed using the Eysenck Personality Questionnaire (EPQ), which is a 25-item self-report inventory that measures the personality traits of extraversion and neuroticism. The questionnaire comprises 14 neuroticism items, which measure the emotional dysfunction of an individual, and 11 extraversion items, which measure the sociability of an individual. The Chinese-language version of the EPQ has demonstrated a high internal consistency of 0.90 [23].

Symptom profiles were assessed using the Chinese Health Questionnaire (CHQ). The CHQ is a 12-item screening instrument that is used to identify minor psychiatric disorders in individuals in the community or in nonpsychiatric departments. The CHQ includes assessment of anxiety and depression. Cheng et al. [24] have demonstrated an internal consistency of 0.79 for the Chinese-language version of the CHQ.

The study protocol was approved by the Institutional Review Board of Kaohsiung Armed Forces General Hospital in southern Taiwan. Informed consent was obtained from all participants before enrollment.

### DNA Extraction

Genomic DNA was extracted from 3.5 ml of peripheral blood from each participant using a QIAamp DNA Blood Mini Kit (QIAGEN, Valencia, USA), in accordance with the manufacturer's protocol. The extracted DNA was stored at -70°C until further use.

### Genotyping

Amplification by the polymerase chain reaction (PCR) was performed in a final volume of 25 μl, which contained 50-100 ng of DNA, 50 mM KCl, 1.5 mM MgCl_2_, 200 μM each of dATP, dCTP, and dTTP, 50 μM dGTP, 150 μM 7-deaza-dGTP, 10 pmol each of two primers (MAOA-F: 5'-ACAGCCTCGCCGTGGAGAAG-3' and MAOA-R: 5'-GAACGGACGCTCCATTCGGA-3'), and 1 U of Taq polymerase (Biolab USA Inc, San Francisco, USA). The PCR was performed on a Hybaid PXE Thermal Cycler (Thermo Fisher Scientific, Barrington, USA) with the following cycling conditions: 35 cycles of denaturation at 94°C for 30 s, annealing at 55°C for 30 s, and extension at 72°C for 30 s. The PCR products were subjected to gel electrophoresis on a 2.5% agarose gel (Amresco, Ohio, USA) with 0.5× TBE running buffer. The gels were stained with ethidium bromide and visualized using an ImageMaster VDS gel documentation system (Pharmacia Biotech, Uppsala, Sweden). The MAOA promoter uVNTR polymorphism was interpreted on the basis of the results of our previous study [25], in which the sizes of the PCR products for the 2R, 3R, 4R, and 5R allelic variants were 320 bp, 350 bp, 380 bp, and 410 bp, respectively. The 2R and 3R allelic variants were defined as short-form variants, and the remaining variants, with a repeat number greater than three, were defined as long-form variants.

### Statistical Analysis

In the descriptive analysis, continuous variables were expressed as the mean ± SD and categorical variables were shown as proportions. We used the algorithm developed by Guo and Thompson [26], which allows an exact test to be performed for traits that are encoded by multiple alleles, to test whether the allelic frequency of the MAOA uVNTR polymorphism in females was in Hardy-Weinberg equilibrium. The Pearson's chi-square test was applied to distinguish differences among the four groups studied. Moreover, the allelic variants were categorized further into two groups according to their transcriptional activity, as described by Sabol et al. [14]. Genotypes that were homozygous and/or hemizygous for the 3R allele formed the low activity group, whereas those homozygous and/or hemizygous for the 4R allele were assigned to the high activity group. Pearson's chi-square test was used to determine whether intergroup differences in allelic frequency between the 3R and 4R variants were significant. Binary logistic regression analysis was carried out to clarify which factors among MAOA uVNTR polymorphism, age, personality traits, gender, smoking, parental attachment, and mental health condition were associated with either suicide attempts in patients with MDD or depressive symptoms in individuals who had attempted suicide. P values less than 0.05 were considered statistically significant. These statistical analyses were carried out using the SPSS 17.0 software for Windows. SEM was performed using the AMOS 7.0 software for Windows to illustrate the interrelationships between the variables studied, which included personality scores, depression, anxiety, suicidal behavior, MDD, and the presence of the MAOA 3R allele. The criteria used to indicate that the null hypothesis model corresponded to the true structure were p values greater than 0.05 and adjusted goodness-of-fit index (AGFI) greater than 0.9.

## Results

We detected the following MAOA uVNTR allelic variants among the participants: 2R, 3R, 4R, and 5R. We did not identify any 3.5R alleles among the subjects. The allelic frequencies and genotype distribution for the MAOA uVNTR polymorphism are shown by gender and group in Table 1. The frequency of the 2R and 5R alleles was extremely low in each group. Three females with MDD carried at least one 2R allele and three community control females harbored one 5R allele. Among the male participants, there were two carriers of the 2R allele in each group, with the exception of the community control group. Only one carrier of the 5R allele, in the community control group, was found among the male participants. The 2R and 5R alleles were not included in subsequent analyses owing to their low frequencies in our studied cohorts. Among the female community controls, the genotype distribution of the MAOA uVNTR polymorphism was in Hardy-Weinberg equilibrium (p = 0.367, data not shown). Among the female participants in each group, the most frequent genotype was 3R/4R, followed by 4R/4R and 3R/3R. The 3R allele was frequent in the male community controls (60.98%) and community subjects who had attempted suicide (52.78%), but not in the other two groups. In contrast, the frequency of the 4R allele was significantly higher in males with MDD than in male community controls (χ^2 ^= 4.182, p = 0.041). Likewise, the 4R allele was more frequent in male subjects with MDD who had attempted suicide than in male community controls (p = 0.170). This finding suggested that the high activity variant (4R) of the MAOA uVNTR polymorphism was more prevalent in male patients with MDD than in those without MDD. This might indicate an association between the 4R allele and depression.

**Table 1:** Allelic frequencies and genotype distribution of the MAOA uVNTR polymorphism in the four groups of participants, namely, community controls, subjects from the community who had attempted suicide, and patients with major depressive disorder who had or had not attempted suicide

			Genotype distribution, N (%)							
			
Group	Age	N (%)	R2/R2	R2/R3	R2/R4	R3/R3	R3/R4	R3/R5	R4/R4	R4/R5
Comm-contl	45.36 ± 13.91	420								
Male		182(43.33%)								
Female		238(56.67%)				88(36.97%)	108(45.38%)	1(0.42%)	38(15.97%)	3(1.26%)
MDD	45.17 ± 15.25	379								
Male		146(38.52%)								
Female		233(61.48%)	1(0.43%)	1(0.43%)	1(0.43%)	81(34.76%)	109(46.78%)		40(17.17%)	
Comm-suic	45.48 ± 20.53	74								
Male		36(48.65%)								
Female		38(51.35%)				11(28.95%)	22(57.89%)		5(15.16%)	
MDD-suic	39.73 ± 14.71	104								
Male		46(44.23%)								
Female		58(55.77%)				24(41.38%)	26(44.83%)		8(13.79%)	

	Allelic frequency, N (%)				Comm-contl		MDD		Comm-suic	

Group	R2	R3	R4	R5	χ^2^	P	χ^2^	P	χ^2^	P

Comm-contl		396(60.18%)	257(39.06%)	5(0.76%)						
Male		111(60.98%)	70(38.46%)	1(0.54%)						
Female		285(59.87%)	187(39.29%)	4(0.84%)						
MDD	6(0.98%)	344(56.21%)	262(42.81%)		1.950	0.163				
Male	2(1.37%)	72(48.32%)	72(48.32%)		4.182	0.041*				
Female	4(0.86%)	272(58.37%)	190(40.77%)		0.220	0.639				
Comm-suic	2(1.79%)	63(56.25%)	47(41.96%)		0.446	0.504	0.010	0.921		
Male	2(5.55%)	19(52.78%)	15(41.67%)		0.355	0.551	0.381	0.537		
Female		44(57.89%)	32(42.11%)		0.169	0.681	0.026	0.872		
MDD-suic	2(1.23%)	96(59.26%)	64(39.51%)		0.022	0.881	0.542	0.462	0.200	0.655
Male	2(4.34%)	22(47.83%)	22(47.83%)		1.879	0.170	0.000	1.000	0.266	0.606
Female		74(63.79%)	42(36.21%)		0.456	0.500	0.934	0.334	0.674	0.412

comm-contl: community controls; MDD: subjects with major depressive disorder; Comm-suic: community subjects who had attempted suicide; MDD-suic: subjects with major depressive disorder who had attempted suicide; N: the number of genotyped subjects; χ^2^: the chi-squared value from Pearson's uncorrected method; asterisk indicates statistical significance. The p-value was obtained by comparing the frequencies of R3 and R4 in each pair of groups; the frequencies of R2 and R5 were ignored because they were low in our population. Overall, the female-to-male ratio was 1.385 (568/410).

On the basis of some previous studies [27,28], we subdivided the allelic variants further into dichotomous groups. The high activity group comprised female homozygotes for the 4R allele and male hemizygotes for 4R, whereas the low activity group contained carriers of the 3R allele. The genotype distributions of the subjects who were homozygous or hemizygous for 3R vs. those who were homozygous or hemizygous for 4R in the four groups studied are shown in Table 2. The high activity variant occurred more frequently among the male MDD and male MDD subjects who had attempted suicide (50% in both groups) than among the males in the other groups (38.68-44.12%). In addition, a significant difference with respect to the frequency of the 4R allele was found between male patients with MDD and male community controls, but no significant differences were found for the other pairs of groups. Given that the frequency of hemizygosity for the 4R allele was the same in male patients with MDD and in male MDD-suicide patients (50% vs. 50%), it was unlikely that hemizygosity for the 4R allele was associated with attempted suicide in male patients with MDD. This finding was basically consistent with the data that showed that male subjects who were hemizygous for the 4R allele had a 1.586-times higher risk of developing depression than male subjects who were hemizygous for the 3R allele.

**Table 2:** The genotype distributions of homozygotes or hemizygotes for the 3R and 4R alleles of the MAOA uVNTR in the four groups of participants

	Genotype, N (%)		Comm-contl		MDD		Comm-suic			
	
Group	3R-H	4R-H	**χ**^**2**^	P	**χ**^**2**^	P	**χ**^**2**^	P	OR	**95% C.I**.
Comm-contl	199(64.82%)	108(35.18%)								
Male	111(61.32%)	70(38.68%)								
Female	88(69.84%)	38(30.16%)								
MDD	153(57.74%)	112(42.26%)	3.016	0.082						
Male	72(50.00%)	72(50.00%)	4.182	0.041*					1.586	1.019-2.467
Female	81(66.94%)	40(33.06%)	0.240	0.624						
Comm-suic	30(60.00%)	20(40.00%)	0.434	0.510						
Male	19(55.88%)	15(44.12%)	0.355	0.551	0.381	0.537				
Female	11(68.75%)	5(31.25%)	0.008	0.929	0.021	0.885				
MDD-suic	46(60.53%)	30(39.47%)	0.487	0.485						
Male	22(50.00%)	22(50.00%)	1.879	0.170	0.000	1.000	0.266	0.606		
Female	24(75.00%)	8(25.00%)	0.329	0.566	0.763	0.382	0.211	0.646		

3R-H: homozygous or hemizygous for the 3-repeat allele; 4R-H: homozygous or hemizygous for the 4-repeat allele; OR: relative odds ratio; 95% C.I.: 95% confidence interval; a: odds ratio of 4R-H to 3R-H in relation to depression in male subjects.

Logistic regression was used to investigate the group of patients with MDD to determine which factors increased the risk of suicide: subjects with MDD who had attempted suicide were compared with subjects with MDD who had not. The results showed that subjects with MDD who were younger in age, more neurotic or who smoked had an increased risk of suicide (β = -0.04, p = 0.002; β = 0.15, p = 0.017; β = 0.79, p = 0.031, respectively), as shown in Table 3. Differences in parental attachment, personality characteristics, mental health condition, demographics, and allelic distribution were also analyzed between the two groups who had attempted suicide, those with MDD and those without. The results showed that, for the two groups who had attempted suicide, subjects who were younger in age, had received more paternal care, and displayed more somatic symptoms were more likely to be in the MDD group than subjects who did not display these characteristics (β = -0.11, p < 0.001; β = 0.15, p = 0.026; β = 1.11, p < 0.001, respectively), as shown in Table 4.

**Table 3:** Logistic regression results for parental attachment, personality characteristics, demographic variables, and allelic frequencies that increase the risk of suicide in patients with major depressive disorder (MDD)

	B	**S.E**.	df	P-value	Exp(B)	95% CI	
						
						lower	upper
Maternal care	-0.05	0.03	1	0.123	1.05	1.00	1.15
Maternal protection	-0.07	0.04	1	0.134	1.07	1.00	1.19
Paternal care	-0.02	0.03	1	0.604	1.02	0.96	1.10
Paternal protection	-0.03	0.04	1	0.481	1.03	0.96	1.12
Extraversion	0.08	0.05	1	0.134	0.93	0.84	1.02
Neuroticism	0.15	0.06	1	0.017	0.86	0.75	0.97
Sex	0.53	0.39	1	0.174	0.58	0.27	1.03
Age	-0.04	0.01	1	0.002	1.04	1.02	1.08
Smoke	0.79	0.37	1	0.03	0.45	0.23	1.02
MAOA uVNTR	-0.06	0.37	1	0.881	1.06	0.60	2.79

Dependent variable: Group- 1: MDD with suicide attempt, 0: MDD without suicide attempt; MAOA uVNTR: 1: 3R, 0: 4R

**Table 4:** Logistic regression results for parental attachment, personality characteristics, demographic variables, and allelic frequencies that increase the risk of major depressive disorder (MDD) in participants who had attempted suicide

	B	**S.E**.	df	P-value	Exp(B)	95% CI	
						
						lower	upper
Maternal care	-0.05	0.04	1	0.246	1.05	0.97	1.14
Maternal protection	-0.10	0.06	1	0.081	1.10	0.99	1.23
Paternal care	-0.06	0.04	1	0.128	1.07	0.98	1.15
Paternal protection	-0.05	0.44	1	0.290	1.05	0.96	1.14
Extraversion	-0.02	0.06	1	0.757	1.02	0.90	1.16
Neuroticism	0.08	0.08	1	0.283	0.92	0.80	1.07
Somatic symptoms	0.25	0.16	1	0.124	0.78	0.57	1.07
Sex	0.60	0.45	1	0.189	0.55	0.23	1.34
Age	-0.06	0.02	1	<0.001	1.06	1.03	1.09
Smoke	0.71	0.44	1	0.11	0.49	0.21	1.17

Dependent variable: Group- 1: MDD with suicide attempt, 0: community with suicide attempt

**Figure 1:**
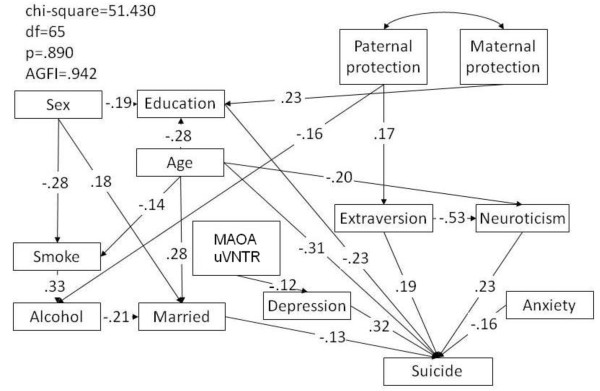
**Parsimonious structural equation model of the comparison between patients with major depressive disorder (MDD) who had or had not attempted suicide**. AGFI: adjusted goodness-of-fit. Dummy variables for suicide: 1- MDD with suicide attempt, 0- MDD patients without suicide attempt

SEM was used to investigate the pathway relationships and to compare the factors of parental attachment, personality characteristics, mental health condition, demographics, and allelic distribution of the MAOA uVNTR polymorphism between the two groups of patients with MDD, those who had made a suicide attempt and those who had not. The parsimonious model showed a good fit, with a p value of 0.890 (greater than 0.05) and an AGFI of 0.942 (greater than 0.9), as shown in Figure 1. The 4R allele of the MAOA uVNTR (0 referred to 4R and 1 referred to 3R in the SEM) did not have a direct effect on attempted suicide in subjects with MDD, but it did have an indirect effect through the mediating factor of depressive symptoms (MAOA-depression: β = -0.12, p = 0.031; depression-suicide: β = 0.32, p < 0.001). Other factors that affected suicide attempts in patients with MDD directly included the demographic factors of level of education, age, and marital status, the personality characteristics of extraversion and neuroticism, and anxiety symptoms. Those patients with MDD who had a lower level of education (β = -0.23, p < 0.001) or younger age (β = -0.31, p < 0.001), were single (β = -0.13, p = 0.053), more extraverted or more neurotic (β = 0.19, p = 0.008; β = 0.22, p = 0.002), or had fewer anxiety symptoms (β = -0.16, p = 0.045) had higher risks of attempting suicide than their counterparts.

**Figure 2:**
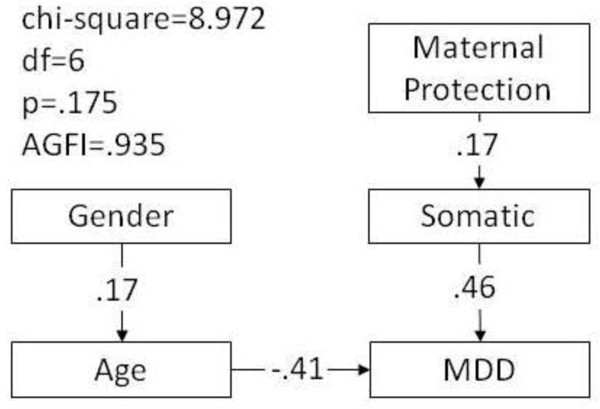
**Parsimonious structural equation model of the comparison between participants who had attempted suicide with and without major depressive disorder (MDD)**. AGFI: adjusted goodness-of-fit. Dummy variables for suicide: 1- MDD with suicide attempt, 0- community participant with suicide attempt

The parsimonious SEM model that compared the two groups of people who had attempted suicide, namely those with MDD and those without, resulted in a good fit with a p-value of 0.175 and an AGFI of 0.935, as shown in Figure 2. The factors that affected directly whether the participants who had attempted suicide had MDD or not were age and somatic symptoms: patients who had made a suicide attempt and had MDD were younger and showed more somatic symptoms than those without MDD (β = -0.41, p < 0.001; β = 0.46, p < 0.001, respectively).

## Discussion

The results of our study showed that the MAOA 4R allele was associated with increased vulnerability to depression among the male subjects in our studied cohorts. This association was not detected in the logistic regression analysis. However, in SEM, the 4R allele of MAOA uVNTR was found to have an indirect effect on suicide attempts in patients with MDD through the mediating factor of depressive symptoms. Other than the length of the MAOA uVNTR polymorphism, additional factors that were associated with attempted suicide in MDD patients included the demographic factors of level of education, age, and marital status, the personality characteristics of extraversion and neuroticism, and anxiety symptoms. By contrast, among those who had attempted suicide, age and somatic symptoms were associated with depression.

In the present study, we detected four different alleles of the MAOA promoter uVNTR polymorphism (2R, 3R, 4R, and 5R), but we did not detect the 3.5R allele in our sample. It has been reported that the 3R and 4R alleles are the most common alleles among different ethnic populations [14]. In our population of 1022 participants, 71.29% of the participants in the community control group were found to carry at least one 3R allele, and 50.69% carried at least one 4R allele. By contrast, only nine participants carried the 2R allele and four carried the 5R allele. The allelic frequencies of the MAOA uVNTR polymorphism in our population were consistent with those described previously [14,19]. The 2R and 5R alleles were rare MAOA alleles in our populations; however, the frequency of the 2R allele seemed to be higher than that in Japanese and Caucasian people (0.2-1.3% vs. none), whereas the 5R allele was less common than in Caucasians (0.2-0.76% vs. 1.6%). Interestingly, in this study, the 2R allele was detected in all groups except the community controls, whereas the 5R allele was observed only in the community control group. Experimental evidence from a cell-based assay, which contradicted the results from Sabol et al. and Deckert et al. [14,15], showed that the 2R and 5R alleles promote higher levels of luciferase reporter gene activity in two human cell lines (IMR-32 and Hela) than the other alleles [29]. Therefore, these two MAOA alleles might be involved in the biological functionality of the MAOA enzyme and possibly might be associated with disease, even though their frequencies are low.

The high activity 4R MAOA allele was found to be associated with depression in male subjects. This was partly consistent with the findings of a previous study by Du et al. [16]. In addition, males who were hemizygous for 4R were at higher risk (OR = 1.586) of developing depressive symptoms than males who were hemizygous for 3R. We suspected that depressed males who carry the 4R allele might also be at higher risk of developing suicidal behavior than those who carry the 3R allele. Subjects who are homozygous/hemizygous for the MAOA 4R allele are assumed to have excess MAOA activity owing to increased transcription, which might result in abnormal serotonin metabolism in the CNS. Furthermore, the 4R allele of MAOA has been linked to high levels of aggressiveness and impulsivity, as well as poor responsiveness to serotonin in the CNS [18]. Thus, the higher risk of suicidal ideation or suicide attempts in subjects who harbor a high activity MAOA allele might be due to a high level of aggressiveness and impulsivity, and a low responsiveness to serotonin, in these individuals. Moreover, the MAOA uVNTR polymorphism has been recognized as one of four serotonin-related SNPs that might affect the decision-making ability of individuals, and thus modulate vulnerability to suicide [30].

From the intergroup comparisons, we found that hemizygosity for the 4R allele was more common in depressed males, irrespective of whether they had attempted suicide. Thus, this result provides further evidence that the 4R allele can modulate the risk for depression, suicide or both by influencing monoaminergic signaling in a gender-specific manner. In addition, as mentioned above, it has been suggested that the high-activity MAOA allele is linked directly to high levels of aggression and impulsivity and low levels of responsiveness to serotonin in males without MDD. Therefore, we speculate that serotonergic signaling might be abnormal in male carriers of the 4R allele who are depressed. This abnormal signaling might result in a phenotype of aggression and impulsivity as a consequence of multifactorial interactions and play a role as risk factor for a suicide attempt. The logistic regression analysis that we carried out confirmed neither a significant association between the MAOA 4R allele and suicide nor an association between the MAOA 4R allele and depression. However, the results showed that subjects who were younger, more neurotic or who smoked had an increased risk of suicide. In addition, subjects who were younger, had received more paternal care, and had more somatic symptoms were more likely to be in the MDD group than their counterparts. This finding reveals the complexity of the involvement of the MAOA 4R allele in vulnerability to a suicide attempt or depression. As a consequence, SEM analysis was used to clarify the role of the 4R allele in the modulation of the risk of suicide and/or depression further, as well as to illustrate the interacting paths between the factors that were possibly associated.

Previous studies have shown inconsistencies with respect to the association between MAOA and suicide. Ono and colleagues [31] and Kunugi and colleagues [32] both failed to find an association between the MAOA polymorphism and suicide. However, Du et al. [16] showed that the high activity allele of MAOA was associated with depression-related suicide in male subjects, and indicated that the risk of a suicide attempt was 3.1 times greater among carriers of the high activity allele than among noncarriers. Our results were also consistent with the results from another study, which investigated Han Chinese subjects in Taiwan [33]. However, we only found borderline significance (p = 0.041) for the association between the 4R allele and MDD in male subjects on the basis of the direct observation of allelic frequencies. However, using SEM, we further determined that the MAOA 4R allele might be associated with suicide among patients with depression after controlling for other associated factors, such as gender, age, the personality characteristics of extraversion and neuroticism, and anxiety symptoms. The results indicated that the 4R allele has an indirect effect on suicide through the mediating effect of depressive symptoms. This effect was not detected when the subjects with MDD who had attempted suicide were compared with the subjects from the community who had attempted suicide. We rationalized that the indirect effect of the 4R allele on suicide was due to the relatively high frequency of the 4R allele among the patients with MDD. Therefore, it might suggest that MAOA 4R allele was associated with enhanced risk of suicidal behavior among those MDD subjects. In contrast to our finding that the MAOA 4R allele was associated with suicide attempts in male patients with depression, the 4R allele was reported to be associated with MDD in German females [34]. The 4R allele was also reported to occur at a significantly higher frequency in female Chinese subjects with MDD than in controls [35]. In addition, the high activity allele was found at a significantly higher frequency in female subjects affected by mood disorders who presented a seasonal pattern or psychotic symptoms in their episodes than in individuals who did not show these characteristics [36]. Given that we found that the frequencies of the MAOA 4R and 3R alleles differed significantly between depressed males and male community controls, we conclude that carriage of the 4R allele by depressed male patients might make them more likely to attempt suicide than carriers of the 3R allele. Our results might explain why inaccurate diagnostic grouping, the lack of investigation of interaction effects, and uncontrolled potential confounding factors might yield inconsistent results when the intergroup differences in MAOA allelic frequencies are simply being observed by using a case-control experimental design.

Comparison of the two groups of patients with depression, those who had attempted suicide and those who had not, determined that, in addition to the MAOA 4R allele, depressive symptoms, lower level of education, younger age, and being more extraverted and more neurotic increased the risk of suicidal behavior. This result was consistent with the findings of a previous study, in which depressive symptoms were shown to be an important predictor for suicidal ideation in groups of patients with psychiatric or general medical conditions, but not in community controls [2]. Another study investigated subjects with MDD, and the severity of depression was identified as a predictor of suicidal ideation [37]. Several studies have shown that personality traits might be related to attempted suicide; for example, neuroticism shows a positive association [4,5]. The SEM model for subjects with and without MDD who had attempted suicide was also applied to the community participants (Figure 2); depression was not found to affect suicidal behavior in the community group. However, the SEM results showed that, among the subjects who had attempted suicide, younger subjects, together with those who had received more paternal care and displayed more somatic symptoms, were more likely to be in the MDD group. Taken together, these findings were consistent with our previous work, which showed that the risk factors for suicidal behavior differed between psychiatric patients and community subjects [2]. Thus, the hypothesis that depressive symptoms are associated with suicidal behavior in the general population is not supported by our study.

Furthermore, our study found an inverse relationship between anxiety and suicide. This is inconsistent with some previous studies which have found an association between anxiety and suicide [38]. However, other studies have also found otherwise [39,40]. Fawcett et al. [41] found that although anxiety was associated with suicide within the first year, but not with 2-10 years. Thus, different subtypes of MDD may be related to suicide. For instance, Grunebaum et al. [39] found the self-blame factor was related to suicide, but not anxiety measured using Hamilton Depression Rating Scales. One of the limitations of this study is that we used the CHQ, which is only a general measure of psychiatric symptoms. Thus we were unable to identify the different subtypes of anxiety which may have different effect on suicide behavior.

In the present study, we analyzed various factors that are associated with suicide, such as cigarette smoking and alcohol consumption, for intergroup differences. A statistically significant difference was found between the two groups of patients with MDD, those who had attempted suicide and those who had not, with respect to cigarette smoking: the participants with MDD who had attempted suicide smoked more cigarettes and drank more alcohol than those who had not attempted suicide. Similar findings have been reported for college students [4,6] and patients with bipolar disorder [42]. According to previous studies, alcohol abuse, drug use, or smoking induces an increase in aggressive or impulsive behavior [42,43]. In a national comorbidity survey panel study, smoking was related to suicidal ideation or suicide attempts; however, it is likely that these associations are not related directly to the causal effects of smoking [44]. In fact, many genetic factors and behavioral traits have been shown to contribute to nicotine dependence in complex ways [45]. It has been documented that the MAOA 4R allele influences the smoking habits of females, as well as nicotine dependence and smoking initiation for male smokers in a Japanese population [46]. By contrast, in Chinese males, carriers of the 3R allele were reported to have a significantly increased risk of smoking as compared with carriers of the 4R allele [47]. More recently, it has been demonstrated that smoking decreases the methylation of the MAOA promoter reliably when DNA extracted from lymphoblasts and whole blood samples is assayed [48]. This finding raises another issue: whether a possible interaction between smoking and the MAOA 4R allele affects not only nicotine dependence but also suicide in patients with depression through a decrease in MAOA methylation and the resultant gain-of-function effect. Further studies that are aimed at delineating the interrelationships among genotype-specific methylation, smoking history, current smoking status, gender, and MAOA promoter uVNTR are warranted.

The results of our study demonstrated that the MAOA 4R allele indirectly confers vulnerability to suicide in male patients with depression. We also produced a parsimonious model of the interrelationships among the factors that were associated with attempted suicide. However, our results should be viewed in the light of some experimental limitations. First, we did not investigate polymorphisms in other genes that are involved in the serotonergic pathway. The lack of this genetic information for data stratification might lead to bias, because the allelic variants of the MAOA uVNTR polymorphism are correlated with normal functionality of serotonin in the CNS. Second, the MAOA uVNTR variants are well known to be associated with different mental symptoms, and the genetic effect varies with gender. We performed data stratification using gender as a covariate. However, data stratification can result in false positive or false negative findings. Third, the community controls did not undergo a medical interview to assess their family history of psychiatric disorders; the required information was obtained simply from self-reports of the participants. Thus, the information gathered might possibly be incorrect. Fourth, survival effects might have influenced our results. However, individuals who had died as a result of suicide could not have been studied, and parasuicidal persons might have been omitted from the present study.

## Conclusion

In conclusion, we have demonstrated that the 4R allele of the MAOA uVNTR polymorphism is associated with enhanced vulnerability to suicide in depressed males, but not in community subjects. Cross-sectional comparisons of nongenetic factors revealed that age, paternal care, and somatic symptoms are associated with MDD, whereas the severity of depression, personality traits (neuroticism, extraversion), age, marital status, and anxiety symptoms were associated with suicide in patients with depression. However, depression did not affect suicidal behavior in the community group. Further large-scale studies are needed to verify the psychopathology of the relationships among the MAOA polymorphism, symptom profiles, including different subtypes of MDD, and attempts at suicide.

## Competing interests

The authors declare that they have no competing interests.

## Authors' contributions

All authors contributed to the design of the study. FW participated in the design of the study, performed the statistical analysis, and drafted the manuscript. DS participated in the design of the study, collected the data, and helped to draft the manuscript. MF carried out the molecular genetic studies and helped to draft the manuscript. MB conceived the study, participated in its design, and drafted the manuscript. All authors have reviewed and approved the manuscript.

## Pre-publication history

The pre-publication history for this paper can be accessed here:

http://www.biomedcentral.com/1471-2350/12/74/prepub
